# EU Cohesion Policy and spatial economic growth: trajectories in economic thought

**DOI:** 10.1080/09654313.2019.1709416

**Published:** 2020-01-04

**Authors:** Daniel Rauhut, Alois Humer

**Affiliations:** a Karelian Institute, University of Eastern Finland, Joensuu, Finland; b Austrian Academy of Sciences, Vienna, Austria

**Keywords:** Economic growth, cohesion policy, regional development, cities, growth pole theory

## Abstract

This paper aims at identifying the current main economic thought influencing the EU Cohesion Policy. Postulates and assumptions on how economic growth spreads spatially in key EU policy framework documents are discussed and compared to different economic theories. Strategic EU documents increasingly foster the urban dimension, and focus resources on cities at the expense of cohesive regional development. The findings indicate large overlaps with Perroux’ ‘growth pole theory’. However, several of the key assumptions of growth pole theory are not met in the new context of post-industrial globalized service economy, which is fundamentally different from its original use. This is a troublesome finding when seen from a strategic planning perspective. Current implications for regional policy and planning boil down to the cardinal question of supporting urban areas and/or peripheries. Taking the strategic EU policy documents and their trajectories in economic thought into consideration, this paper confirms that regional development focuses on cities. Yet, it suggests a new perspective on an urban-centred EU Cohesion Policy, one that normatively requests the ‘responsibility’ of cities towards their hinterland, instead of fostering a further dissociation of cities from their hinterland. This suggests a reorientation towards supporting the linkages between urban areas and peripheries.

## 1. Introduction

Over the last 10 years, the EU Cohesion Policy has changed its rhetoric and focus from promoting a balanced development between regions in Europe, to pronouncing the role of cities, stimulating global competitiveness in cities linked to an emerging urban agenda and a Europeanisation of urban policy (Atkinson, 2015; Avdikos & Chardas, 2016; Chilla, 2012; Dukes, 2008; Purkarthofer, 2019; van den Berg, Braun, van der Meer, & Mingardo, 2007). Today, EU policy objectives aim to enhance regions through the improvement of their cities, and urban competitive capacities in the world economy. The policy objectives highlight a large number of strategic suggestions, recommendations, methodologies and substantive policies. These include general economic development strategies (e.g. stressing competitiveness based on a knowledge economy), spatial models (e.g. polycentrism), priority territorial elements and actions (e.g. cities as engines of growth), and the importance of brown-field site rehabilitation or specific performances (e.g. accessibility to services or infrastructure) (Faludi, Stead, & Humer, 2015; Capello, Caragliu, & Fratesi, 2015; Camagni & Capello, 2015a). Overall, cities are assumed the drivers of economic growth for the whole territory (van Oort, de Geus, & Dogaru, 2015; Gordon, 2013; Rauhut & Hatti, 2017).

A key postulate is that economic growth and development will spread from metropolitan areas and city regions, through small and medium sized towns, to rural and lagging regions. In the process, larger cities should be encouraged to network and cooperate with smaller cities and their surrounding territories in order to spread a positive economic development territorially downwards. This form of development can be stimulated by implementing more urban friendly policies, and polycentric development plays an important role in this process (CEC, 2017a; European Union, 2007, 2011, 2016).

The perspective that economic growth in the cities will spread to rural and peripheral areas differs, dependent on what growth theory is applied. In general, size and economic diversification will lead to increased productivity and economic growth. Economies of scale arise due to size, diversification, and the knowledge spillover between companies and sectors. In *urbanisation economies*, agglomeration effects will occur due to higher productivity, which is a function of the geographic concentration of people and capital (Jacobs, 1984; Bairoch, 1988; Krugman, 1993; Quigley, 1998). *Localisation economies* argue that agglomeration effects relate back to the positioning of companies with similar production requirements to a certain geographic area. In such locations, clusters and specialization will likely be the drivers of productivity growth (Lucas, 1988; Romer, 1994; Arrow, 1962; Marshall, 1890).

This paper aims at identifying the current main economic thought influencing the EU Cohesion Policy by discussing the assumptions and postulates of spatial economic growth, which are expressed in key policy documents. Specifically, the causalities between how policies stimulating cities and agglomerations lead to development and growth in peripheral and lagging regions will be examined. Two research questions will be answered: (1) How does the EU consider spatial economic growth to emerge? (2) What are the implications for regional policy and planning?

In pursuing these questions, the discussion will identify the *economic thought* influencing the EU Cohesion Policy in key EU policy documents. Notwithstanding this, EU Cohesion Policy is ultimately crafted by actors and their political ideologies as well as their institutional context (Faludi, 2018). Moreover, it must be emphasized that this paper has no ambition to evaluate the efficiency of the Cohesion Policy, nor to discuss potential gaps between the political declarations and the implementation of the Cohesion Policy in the regions. Of interest, however, is the gap between policy declarations featured in key policy documents and theoretical arguments in economic thought.

In order to identify key policy documents, we need to define ‘the EU’. In this paper, activities of the EU Commission and the European Parliament are considered as being undertaken by ‘the EU’. Although the single Member States have diverging and sometimes even contradictory policy agendas, joint policy declarations are also considered as being undertaken by ‘the EU’. Policy declarations, reports and communications from the EU Commission and European Parliament are considered as key policy documents, as are joint policy declarations by the Member States such as e.g. the ESDP, the Territorial Agenda, and Urban Agenda.

In the following section 2 of this paper, we elaborate on the recent key strategic EU policy documents, and develop a combined view of their strategic frameworks as to how economic growth is supposed to spread spatially and which concepts are behind it. Sections 3 and 4 mirror that policy framework and look (back) into their theoretical foundations as well as evidence drawn from past policy practice. In sections 5 and 6 we take a look forward to the implications for the EU Cohesion Policy post 2020, address the ‘cardinal question’ as to whether an urban and/or rural focus should be maintained, as well as identifying future debates and research to be pursued.

## 2. A conceptual framework derived from policy context

The 2007 Territorial Agenda identifies cities as drivers of development and economic growth. By stimulating city networks and a polycentric development, the global competitiveness will be strengthened (European Union, 2007, p. 4).

Cities which function as regional centres should cooperate as parts of a polycentric pattern to ensure their added value for other cities in rural and peripheral areas with specific challenges and needs (e.g. structurally weak parts of islands, coastal zones and mountainous areas). (European Union, 2007, p. 5)

This is developed further in the Territorial Agenda 2020 when stating that the metropolitan areas and city regions are assigned an active obligation to ensure that surrounding regions can benefit from their added value. ‘Metropolitan regions should also be aware that they have responsibility for the development of their wider surroundings’ (European Union, 2011, p. 7). The underlying idea is that economic growth and development will eventually spread from globally competitive cities to regional centres, through city networks and polycentric structures, and from medium-sized cities to small cities, and from there to rural and peripheral areas. Hence, cooperation between city regions as well as between small and medium-sized cities should be stimulated.

The Urban Agenda (European Union, 2016, p. 5) states the need to implement more urban-oriented policies in the EU, justified by the fact that circa 70% of EU population live in cities. The importance of networking and cooperation of cities with their functional areas and surrounding regions is thereby crucial and shall be promoted through a partnership-approach (Purkarthofer, 2019). According to the 7th Cohesion Report, the positive developments in cities will spill-over to the city’s hinterland (CEC, 2017a, p. xii). Since the establishment of the EU structural funds period 2014–2020, smart specialization strategies are an ex-ante condition for every region and member state for being eligible to access European regional development funds (ERDF), preparing a regional innovation-oriented strategy building on e.g. the industrial culture, sectoral clusters and knowledge bases of a region. By arguing for a place-based, tailor-made logic (McCann & Ortega-Argilés, 2015), smart specialization strategies must account for the principle disadvantage of innovation potential of lagging regions concerning core regions.

The Europe 2020 regional strategy describes what should be done to make the EU competitive. However, needed actions are described at EU- and national levels. For some of the ‘Flagship Initiatives’, the Member States should focus on the urban dimension; not much is said about non-urban areas (CEC, 2010). Nor does the *Common Strategic Framework* (CSF) by the Assembly of European Regions bring any clarity in how spatial economic growth will occur or even spread (AER, 2012, p. 9). Already the Green Paper on Territorial Cohesion (CEC, 2008) has been thin on explaining how economic growth spreads spatially, and the recent White Paper on the Future of Europe is silent on this issue (CEC, 2017b) something, which has been noted (Böhme & Toptsidou, 2017).

The European Commission wants to stimulate local and regional development by e.g. integrated territorial investments (ITI) and community-led local development (CLLD). For the ITIs, the Member States design the territorial tools to ensure its implementation according to aims of the national territorial strategies; the implementation of CLLD is based upon the close cooperation and integrated use of the (ERDF/FEDER) ‘Regional Funds’ to deliver local development strategies (CEC, 2018, pp. 15–16). This CEC document also allows intra-national transfers to less developed regions:

to accommodate Member State's needs to tackle specific challenges, Member States should be able to request a transfer from their allocations for more developed regions or for transition regions to less developed regions and should justify that choice. In order to ensure sufficient financial resources for less developed regions, a ceiling should be established for transfers to more developed regions or to transition regions. Transferability of resources between goals should not be possible. (CEC, 2018, p. 21)

Table 1 summarizes the most important recent strategic documents with regard to the urban dimension in a territorial and in an economic growth sense. The table indicates a slightly different take of the role of urban areas depending on the issuing source. Particularly earlier *inter*national documents – i.e. documents agreed on by representatives of the EU Member States – emphasize the urban-rural connections. Only the most recent document, the Urban Agenda, carried forward by the highly urbanized EU Member State of the Netherlands, is purely focused on urban areas. The *supra*national documents – i.e. issued by the European Commission – are more straightforward in declaring urban areas as a key territorial type when it comes to economic growth. Important to mention is the general marginal role of territorial matters within broad EU strategies, such as Europe 2020.

**Table 1.** Recent strategic documents of key importance on EU level and the ‘urban’.

Document	Issued/ratified by	The ‘urban’ in a territorial context	The ‘urban’ in a growth context
Territorial Agenda 2007 European Union (2007)	Informal Ministerial Meeting on Urban Development and Territorial Cohesion (hosted by Germany)	Polycentricity; Urban-rural relation	Cities as drivers of economic growth
Territorial Agenda 2020 European Union (2011)	Informal Ministerial Meeting of Ministers responsible for Spatial Planning and Territorial Development (hosted by Hungary)	Polycentricity; Urban-rural relation	Cities with responsibilities towards the hinterland
Urban Agenda/ Pact of Amsterdam European Union (2016)	Informal Meeting of EU Ministers Responsible for Urban Matters (hosted by the Netherlands)	Urban area as the dominant and decisive territory	Urban areas in a key role to pursue the three Europe 2020 growth objectives
Europe 2020			
CEC (2010)	European Commission	*-generally absent-* Broadly mentioning national, regional and local levels.	*-generally absent-* Broadly about smart, sustainable and inclusive growth
7th Cohesion Report CEC (2017a)	European Commission (DG REGIO)	Urban areas most efficient as well as most challenged	Regions with large cities have highest growth

Source: own elaboration.

From these policy framework- and regulatory documents, we can derive the EU’s conceptual framework on what role cities *should* play in regional development, and how economic growth spreads spatially. Overall, more urban friendly policies, stimulating economic activities in urban areas will stimulate a positive economic development for all types of regions. (1) Economic growth and development will trickle down from bigger cities, to medium-sized cities, to smaller cities, and cascade to towns within polycentric urban regions. (2) The economic growth in the polycentric urban regions will spill over to the hinterland of these city regions or big cities, to peripheral and remote areas. (3) Polycentrism will decrease the gap between economically strong and expanding urban regions vis-à-vis the peripheral regions. (4) The bigger cities and urban polycentric regions have a responsibility to develop their hinterland. While the first three points represent theoretical and descriptive expectations, the fourth formulates a normative expectation of how cities should behave, so that economic growth will spread spatially.

Similar conclusions on how the EU frames spatial economic growth has been identified previously (Romanian Regional Development Program, 2013; Bere & Silvestru, 2015; Bere, Precup, & Silvestru, 2015; Komarovskiy & Bondaruk, 2013; Christofakis & Papadaskalopoulos, 2011; Benedek, 2016). It is also worth noting that several ESPON projects have their point of departure in describing how the EU frames spatial economic growth (ESPON, 2010, 2012, 2014). These studies even give a name to the theory by which the EU frames its spatial economic growth: the *Growth Pole Theory*. Although several key policy documents are silent on spatial economic growth, Zaucha, Komornicki, Böhme, Swiatek, and Zuber (2014) suggest growth poles to bridge the Territorial Agenda and the Europe 2020 strategy.

## 3. Trajectories in economic thought

Without doubt, as discussed in the section above, the trajectories on how the EU considers economic growth to spread spatially, resembles the old arguments of *Growth Poles Theory*. Francois Perroux (1950) launched the idea of growth poles in 1949 at a Harvard lecture. The central idea of growth poles theory is that economic development or growth is not uniform over an entire region, but instead takes place around a specific pole (or cluster). This pole is often characterized by core (key) industries around which linked industries develop, mainly through direct and indirect effects. Because of the scale and agglomeration of economies near the growth pole, regional development is unbalanced. At a later stage, the emergence of secondary growth poles is possible, mainly if a secondary industrial sector emerges with its own linked industries, contributing to the regional economic diversity (Darwent, 1969; Perroux, 1970). Hirschman (1958) modified the growth pole theory and stated that inter-regional imbalances in development are inevitable in economic growth. Economic growth in a developed region produces favourable trickle-down effects to lagging region (Parr, 2015).

Through the economies of scale and the importance of agglomeration economies emphasized by Perroux, theories of agglomeration economics become interesting. New Economic Geography theory argues that from a macro-perspective, it is rational to concentrate on central areas. These areas not only create economies of scale, but also have lower transport costs (Krugman, 1993, 1991a, 1991b). In agglomeration economics, clustering and concentration lead to productivity increases and economic growth.

In the discussion on economic growth in the EU, ‘agglomeration’ is seldom heard of, and instead, the term *polycentric regions* is used. While in economic theory, ‘agglomeration’ is in the central vocabulary, geographers and planners frequently refer to ‘polycentricity’ (Rauhut, 2017). Polycentric urban regions are explained with the same rationale as agglomerations, however, potentially on a larger, urban-regional scale, and thus subject to more distributional effects. Generally, polycentric urban regions are seen to provide, or at least aim at accumulating the same favourable economic conditions as urban agglomerations. Hence, they can be used as growth poles (Meijers, Hoogerbrugge, & Cardoso, 2018).

A central argument in growth pole theory is link between the growth centres and their nearer environment. As growth begins in some areas, these places turn into growth poles and growth spreads out to the surrounding areas. In each growth pole there are some driving industries encouraging new industries in supply chains. Growth poles and supply chains are not spread evenly spatially, and hence regional disparities are inevitable (Li, 2012).

Myrdal (1957) identified troublesome side effects of Perroux’s growth poles. Economically expanding regions will attract capital and labour from lagging regions; investments are made in prosperous regions simply because investments have been made there before (cumulative causation), and migrants are in their prime working age with demanded human capital and skills. Lagging regions will further drop behind as the flow of human resources and capital tends to favour the more prosperous regions. In other words, polarization between regions will increase.

There are two central aspects of Perroux and the Growth Pole Theory seldom discussed. According to McKee (2008), Perroux was focused on *economic* space in his theory, not on territorial space. Economic space contains e.g. market shares, distribution and supply chains. This makes Growth Pole Theory a-spatial, which also Parr (2015) notes. Secondly, McKee (2008) emphasizes the fact that Growth Pole Theory focuses on *manufacturing*, and not on the service economy. Modern business services – e.g. financial, insurance, facilitative and support services – are not tied to certain growth poles, but are global in character. Already Wickham (1963) made a similar conclusion when he argued that the ongoing internationalization of the economy would challenge local supply chains and national economic planning. Related to this, we want to point at the fact that global supply chains did not exist when Perroux developed his theory, and we need to keep in mind that global supply chains have challenged several dimensions of growth pole theory as the growth and linkages generated by a core industry may involve activities located elsewhere. This is nowadays valid also for manufacturing.

Moreover, Perroux’s Growth Pole Theory must be placed in a proper context. In the French war-destroyed economy, there was an urgent need to rebuild heavy and manufacturing industry in the late 1940s and the 1950s. The ideas of growth poles and local supply chains fitted well into French political ambitions to rebuild its economy after the war. In the work of rebuilding the French economy, through economic plans, Perroux played a key role (Wickham, 1963).

None of the major works in economic thought and economic growth mention Perroux nor ‘growth pole theory’ (Roll, 1992; Schumpeter, 1994; Blaug, 1997; Backhouse, 1991; Rostow, 1992). The reason why Perroux is not included in these studies may be related to lack of originality or theoretical significance. However, everything related to growth theories is more related to politics than to economics: there is a political acceptance to organize resources to stimulate economic growth in a certain way (Blaug, 1994; McCluskey, 1985). If these efforts are in line with economic theory, or even economic advice, is a completely different question. In line with this argument, Perroux and growth pole theory is more about politics than economics, and it can explain his absence in the major works in economic thought and economic growth.

For those who read Hirschman (1958) they will realize that he focused his research on *development economics*, and his modified growth pole model, with ‘trickle down’ effects to the surrounding areas, aimed at conditions in industrializing and developing South Asia. His contributions to economic theory and economic thought in this area are recognized (Rostow, 1992; Backhouse, 1991). Nevertheless, the context in which Hirschman developed his theory is very different indeed from the twenty-first Century EU with a post-industrial economy dominated by a service sector.

Castells' *city network theory* has claimed a ‘dissociated city hypothesis’, meaning that interconnected cities are globally well networked, while at the same time losing their ties to their immediate hinterland (Castells, 1996). The dissociated city hypothesis applies particularly to cities with a knowledge- and innovation-intensive economy, and reflecting cities that seek to succeed in those sectors. What McKee (2008) concludes on the applicability of growth pole theory in modern services is in line with Castells (1996). Much more fluid global supply chains in a knowledge- and innovation-intensive economy have replaced the regional supply chains of a growth pole in the manufacturing sector. In order to be competitive at a global level, a growth pole may actually have to cut the ties with its hinterland, as suggested by the ‘agglomeration shadow’ argument; that in the shadow of the core city, small hinterland municipalities cannot flourish (Cardoso & Meijers, 2016).

In short, such a process means that, cities further extend their advantages over rural regions, which become peripheral. Kühn (2015, p. 370) reaches a similar conclusion when saying that a ‘growth pole results from the advantages cities have as agglomerations, their density of services and activities. An agglomeration provides a context conducive to innovations, which in turn attracts more activities and reinforces the agglomeration’.

Thus, the meagre theoretical support, not to say the theoretical critique and objections to the applicability of the growth pole theory appears to have had little effect on the formulation of the EU Cohesion Policy and its fostering of knowledge and innovation industries. In line with the arguments of Blaug (1994) and McCluskey (1985), spatial economic growth in EU Cohesion Policy appears to be more about politics than economics.

**Figure 1.**
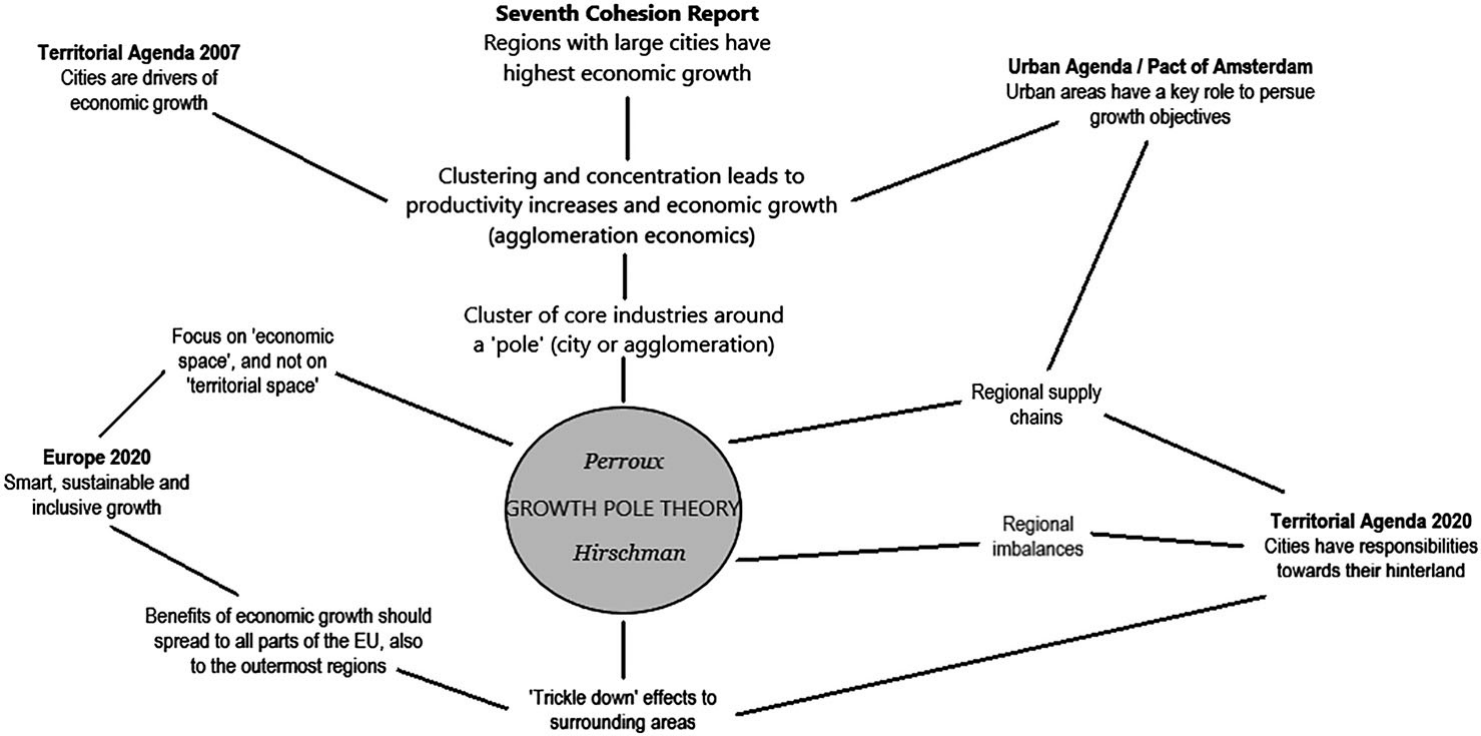
An outline of the links between the Growth Pole Theory and how spatial economic growth are described in recent key EU policy documents.

Figure 1 outlines the trajectories to Perroux and Hirschman on how economic growth is considered to spread spatially in the recent key EU policy documents.

## 4. Has economic growth ‘trickled down’?

One of the few studies analyzing the possible spatial spread of economic growth from urban areas to their surroundings identifies that rural regions near urban agglomerations have experienced positive effects from EU Cohesion Policy for the period of 2000–2006 (Gagliardi & Perocco, 2017). The crisis in 2009 illuminated the fact that crises are spatially unevenly distributed. Not only were certain countries particularly hard hit, but also some regions (Hadjimichalis, 2011). The positive effect of EU Regional Policy spending appears to have been weaker during the crisis, and the convergence aims of the policy became more difficult to attain (Becker, Egger, & Maximilian von Ehrlich, 2018). Accordingly, the positive effects identified by Gagliardi and Perocco (2017) for rural regions near urban agglomerations between 2000 and 2006 may no longer be valid. As Fiaschi, Lavezzi, and Parenti (2017) note, the period of 2000–2006 appears to have been exceptionally successful, but as noted by Becker et al. (2018), the positive effect of cohesion spending is more difficult to identify after the crisis 2008/2009. Ederveen, de Groot, and Nahuis (2006) did not find any impact of Cohesion Policy on economic growth performance, which opposes the findings of Gagliardi and Perocco (2017), and Medeiros (2013, p. 1980) concludes that ‘there are no conclusive agreements on the impacts of EU Cohesion Policy in the existing scientiﬁc literature’. A recent study by Mykhnenko and Wolff (2019, p. 473) opposes this by concluding that the findings in their study shows that EU Cohesion Policy actually stimulates economic growth, and that ‘The beneﬁcial impact of the EU Cohesion Policy can no longer be ignored in this regard’.

Other studies suggest that there is no relationship between urban concentration and economic growth (Frick & Rodríguez-Pose, 2018), and that the significant investments in transport and infrastructure supported by Cohesion Policy have no effect on economic growth (Crescenzi & Rodríguez-Pose, 2012). What appears to impact economic growth the most is the quality of government, the governance that is applied, and institutional factors (Rodriguez-Pose & Garcilazo, 2015; Farole, Rodríguez-Pose, & Storper, 2011). Ederveen et al. (2006) also stress robust institutions as an important precondition for Cohesion Policy to stimulate economic growth.

It may make economic sense for the EU to focus on cities, and this path has been followed since the ESDP was introduced (CEC, 1999). For the hinterlands however, such a focus results in the maintenance or fostering of a dependency-situation (Kühn, 2015). While urbanized NUTS3 regions do well under an urban policy focus, completely non-urban NUTS3 regions are excluded from profiting from urban dynamics (Meijers & Burger, 2017). Additionally, regional governments try to serve the core of their regions and not the non-core parts, hence EU funding does not spread outside of the cores of peripheral regions (Nagy & Benedek, 2018).

The EU Commission policy initiatives ITI and CLLD are partly designed to counteract increasing disparities between remote/peripheral and urban/metropolitan regions. In practice, the ITI is closely linked to the implementation of the urban agenda (Isola, Leone, & Pira, 2017). Recent research also questions the extent that implementation of CLLD can solve structural problems in less developed areas, as it is dependent on the present administrative order and governance in the single Member States (Servillo, 2019).

Ultimately, it can be seen that the link between bigger cities and their hinterlands does not stimulate a spread of economic growth, and it can also be questioned whether economic growth has in fact ‘trickled down’ from the cities in core regions to small towns in non-core and remote regions.

## 5. Implications for post-2020 EU Cohesion and regional policy

EU Cohesion Policy programming is driven by a bureaucratic logic that is primarily concerned about the legal certainty behind programmes, and the correct and efficient input of financial means in the implementation of programmes. However, despite these being right values, this approach may have hampered the implementation of principles of the strategic spatial planning discourse such as those promoted by e.g. Albrechts, Healey, and Kunzmann (2003), Albrechts and Balducci (2013) and Mäntysalo (2013), and have had no influence on the logic of how EU Cohesion Policy is programmed. Significantly, the links between strategic spatial planning and EU Cohesion Policy may be more or less visible, not least depending on the weight of EU Cohesion funding in member states (ESPON, 2018). More importantly in this context is the ‘how’ such policies are implemented – the procedural side. To this end, strategic spatial planning tells that we need daring strategies and programmes that extend beyond blueprint, incremental change and re-iterative ideas, and which consequently urge for a less bureaucratic control mechanism and offer more room for experimentation, even if that means less predictive outcomes and imperfect monitoring.

A strategic spatial planning approach motivates for selective, focused visioning, implying that not all (minor) issues can be comprehensively addressed simultaneously and with the same attention. A significant concern is to have impactful strategies. In this respect, a significant shift of EU Cohesion Policy attention towards urban areas will help to innovate and allow an escape from old beaten tracks. However, outdated theoretical underpinnings behind such a shift may lead to an implementation-gap. In other words, even if it is a welcomed step from a strategic spatial planning perspective, urban-focused policy, as an innovative step in the development of EU Cohesion Policy, may lead to unwanted developments if it employs outdated theories used in a fundamentally different context.

Two major implications can be raised from the theoretical critique of the EU’s conceptual model of spatial economic growth. Firstly, if EU Cohesion Policy acknowledges the argument of polarization theory with a trickle-down effect, then policy intervention in well-off regions or the well-off urban parts of a region alone will not foster convergence. The emphasis on supporting urban areas – or overly employing measures that favour urban areas – is in contradiction to traditional public regional policy. The latter seeks for inner- and inter-regional equal standards of economy and living and for that reason purposively places investment into lagging regions or hinterlands of regions.

Secondly, if the EU Cohesion Policy acknowledges the argument of city network theory and the dissociated city hypothesis, then a further fostering of knowledge and innovation policy will weaken continental urban-rural ties. While cities may develop even better, peripheries will fall further behind. According to dependency theory, when weakening these urban-rural ties, or, weakening the position of rural areas towards urban areas, socio-political tensions will be unavoidable between centre and periphery, leading to various consequences, concerning issues of repression, conflict and replacement, and not always to decentralization (Friedmann, 1972). Indeed, Rodriguez-Pose (2018, p. 190) describes a Western world situation in which ‘[f]uture economic growth and development at country level and in the most prosperous regions is being jeopardized by the revenge of the “places that don’t matter”’, not least expressed through ballot-boxes (Essletzbichler, Disslbacher, & Moser, 2018; Dijkstra, Poelman, & Rodriguez-Pose, 2019). In addition, other scholars have also identified the source of increasing urban/rural societal and economic divide in many Western countries (McCann, 2016), especially when seen in the dominance of urban economics and new economic geography narratives (Rodriguez-Pose, 2018).

A general implication is that, not only with analytical but also normative trajectories, presumably the EU has to decide between pro-*urban* or pro-*lagging* regional policies. Today, a balancing act between the two is still pursued (Nosek, 2017). Farago and Varro (2016) advocate *pro lagging* regions, whereas Medeiros and Rauhut (2018) advocate *pro urban* regions. Continuing arguments for *pro full coverage* approaches (e.g. Camagni & Capello, 2015b) might result in all and nothing, especially when foreseeing tighter EU Regional Policy budgets in future funding periods, together with imperfect funding mechanisms and schemes (Cerqua & Pellegrini, 2018; Dabrowski, 2015). This decision making process should be explicitly communicated. Moreover, the current situation with *urban*-favouring policies accompanied by a *lagging region*-supportive rhetoric does not counter act or even mitigate the current development approach. On the contrary, it aggravates the problems, which is, for example, illustrated by the very uneven capacities and abilities to conduct and employ the obligatory smart specialization strategies, which are inherently more suitable for urban, industrialized, and institutionally thick regions, and institutionally and economically more challenging for peripheral regions (Capello & Kroll, 2016).

Given that convergence across European regions remains the prime objective of EU Cohesion Policy, some attention will have to remain focused on the lagging, peripheral regions. Two alternatives to the current, urban-favouring situation appear plausible. One could be that the EU Cohesion Policy turns into an urban-only policy, and rural areas are principally left to the Common Agricultural Policy (CAP) and DG AGRI’s interventions under the EAFRD fund, like LEADER. A second alternative may be that member states start their own internal programmes with or without EU funding and replace EU policy interventions in rural regions. Notwithstanding this, the latter alternative implies a severe step towards renationalisation and rising inequality between EU member states – given that financially less capable member states might be far from compensating Cohesion Funding in their own rural territories. After all, both alternatives further emphasize EU Cohesion Policy intervention for urban areas, towards cities as economic engines; but also, increased resources could go into urban questions of social inclusion, education and other social urban issues.

There is one more possible avenue – apart of the question of either urban, or rural, or both – that builds on the last, normative, aspect of how EU frames spatial economic growth. The responsibility of urban areas over the development of their hinterland. In this vein, it shall be less concern about which target areas to fund. Anyhow, as we have argued, the market forces of our post-industrial economy are principally more favourable towards urban areas. Investment into stimulating industry in peripheral areas is not efficient. Therefore, in meeting the call for ‘responsibility’, the EU budget might be best invested into fostering a transfer, connection and relation *between* urban and peripheral areas, instead of focusing investment into one of the regional ideal types. Responsibility of a city over its hinterland exceeds mere economic growth targets. Investment then targets on regional functions and services of general interest, or, more broadly, on elements of a ‘foundational economy’ to foster socio-economically ‘grounded cities’ that serve and stabilize their hinterlands (Engelen, Froud, Johal, Salento, & Williams, 2017).

The outlines for an EU Cohesion Policy post 2020 (European Union, 2018a) indicate a further emphasis on innovation-strong smart specialization strategies, leading towards a smarter, carbon-free, digitally connected Europe. Social and sustainability issues are anchored on a local level. From a territorial point of view, this indicates high efforts towards strengthening the urban dimension in Europe. However, exactly what the regional ‘responsibility’ and the connection of cities towards hinterlands and rural areas entails is hardly visible. ‘Rural’ or ‘peripheral’ vocabulary is *completely* absent in the public summary of the legal proposal (European Union, 2018b). In the thematic objective 5 (of 5), the legal proposal speaks of the ‘sustainable and integrated development of urban, rural and coastal areas’ (European Union, 2018a, p. 7), which is simplified in the public summary as ‘sustainable urban development’ (European Union, 2018b, p. 1). This zoom in on a single formulation exemplifies the ambivalent strategic outlook on urban-centred EU Cohesion Policy post 2020, and the regional dimension of an urban-policy is only marginally addressed.

## 6. Concluding remarks

In the key EU policy documents, it is proclaimed that by stimulating economic activities in urban areas, a positive economic development will be stimulated for all types of regions. Economic growth and development will spread from metropolitan areas and city regions, through smaller towns to rural and lagging regions. Polycentrism is assumed to decrease the gap between economically strong and expanding urban regions, vis-à-vis peripheral and lagging regions (European Union, 2007, 2011, 2016; CEC, 2017a). The post-2009 observation that it is difficult to identify the positive effects of the cohesion spending (cf. Becker et al., 2018) may imply that the link between major cities and polycentric urban regions, on one side, and hinterland or rural areas on the other, have become weaker.

This paper analyses the current main economic thought influencing the EU Cohesion Policy by discussing the assumptions and postulates on spatial economic growth expressed in key policy documents. The intended focus is to illuminate the causalities between how friendlier policies for cities and agglomerations will lead to development and growth in peripheral and lagging regions. We have considered two specific research questions.

1. *How does the EU consider spatial economic growth to emerge?* In short, strategic EU documents consider economic growth and development to spread from bigger cities, to medium-sized cities, to smaller cities, and to towns within polycentric urban regions; and from there it will spread to peripheral, remote and lagging areas. The economic growth in the polycentric urban regions will spill over to the hinterland of these city regions or big cities. Polycentrism will decrease the gap between economically strong and expanding urban regions vis-à-vis the peripheral and lagging regions. This ‘cascadic’ view on how economic growth spreads spatially in the EU Cohesion Policy is analogous with the Growth Pole Theory, which Perroux formulated in 1949 to stimulate the rebuilding of the war-destroyed French manufacturing industry. Hirschman contributed in the 1950s with the suggestion of ‘trickle down’ effects to the surrounding area from the growth pole. Hirschman argued that this model could help industrializing poor countries in the developing South Asia. Finally, and this is important from a normative policy stance, the bigger cities and urban polycentric regions have a responsibility to develop their hinterland.
2. *What are the implications for regional policy and planning?* Outdated theories or theories used in fundamentally different contexts from what they were intended to operate in may lead to unwanted effects. It is an innovative policy measure to focus on cities after years of limited success in bridging the gaps between rich and poor regions by focusing on the poorer regions. From a strategic planning discourse, the negative side effects however outnumber the positive ones. Rodriguez-Pose (2018) has identified how the ‘places that don’t matter’ protest against being neglected by the elites in the economic centre. The frown reaction of these places correspond to the increased focus on innovation-strong cities. Another side effect on planning is evident when a theory developed for rebuilding the manufacturing industry in a war-destroyed country, spiced with ideas from the industrializing Third World from the 1950s, will ‘trickle down’ to the surrounding areas: tomorrow’s society is planned by yesterday’s ideas. The needs of a post-industrial economy with a dominating service economy requires a fundamentally different planning strategy than industrializing countries in the Third World or countries that try to rebuild their industry destroyed by war.

Looking ahead, in both economic and social policy respects, there are complicated avenues for the European Union to consider. From an economic growth stance, a further fostering of the urban focus would create the best effects overall, however this would mean a final farewell to positions that seek to decrease and diminish regional (and social) inequalities. Of recent concern are the developments of rising populism and an aspiration for de-globalisation which have arisen in left-behind regions (Essletzbichler et al., 2018; van Bergeijk, 2018), and of note is Rodriguez-Pose (2018, p. 205) who warns that ‘[w]ithdrawing intervention in these areas will inevitably add fuel to the fire’. In this respect, any future decision on focusing increasingly on urban agglomerations or lagging peripheries is not to be seen as a sectoral or regional policy problem, but more represents Europe’s cardinal identity question: Is the European Union finally a single market of global size, or a social union, or obsolete. An answer to that very question will automatically imply the path for urban/regional development in Europe. Maybe, the answer lies in rediscovering the responsibility of the cities over their hinterland. As such, the discussion in this paper can help to establish a future development track that focuses on the responsibility of cities towards their hinterland. So, while normative strategies and policies may increasingly focus on cities and urban areas, this may not be for their sake, but more so for the sake of urban-rural linkages.

The added value of this study is how the trajectories of economic thought in the EU Cohesion Policy are revealed. Of significant importance, the growth pole theory used by the EU to frame its spatial economic growth was founded in 1949 by Perroux to rebuild the French manufacturing industry after WW2, and developed by Hirschman in the 1950s, as a strategy to industrialize countries in South Asia. This context is significantly different from that of the post-industrial European Union, dependent on global supply chains, and with a dominating service sector. This study therefore questions as to what extent this strand of economic theory can increase and improve the economic competitiveness of the European Union from a global perspective. A second added value of this paper is that it suggests a new perspective towards an urban-centred EU cohesion policy, whereby instead of fostering a further dissociation of cities relative to their hinterland, we argue for a reorientation towards supporting *linkages* between urban areas and peripheries.

At least three suggestions for future research can be identified from this study. One deals with the knowledge gap related to why the growth pole theory was included in the EU Cohesion Policy, and interviewing politicians and senior civil servants in the Commission may provide an answer as to what rationale placed growth pole theory on the EU policy agenda. A second topic for future research is the need to explore the weak – or even absent – regional dimension of this city-focused policy as well as possible tensions between two understandings of ‘region’ within future EU Cohesion Policy: a traditional understanding of NUTS regions and an alternative understanding of functional urban regions. Thirdly, the EU Cohesion Policy and spatial planning discourse is surely no monolith, as, for example, Waterhout (2008) or Adams, Cotella, and Nunes (2011) indicate. Thus, the European Commission as well as the (single) EU Member States might pursue various interests concerning an urban centred EU Cohesion Policy.
